# A Practical Guide to Transition State Analysis in Biomolecular Simulations with TS-DAR

**DOI:** 10.1021/acs.jpcb.5c06097

**Published:** 2025-11-18

**Authors:** Eshani C. Goonetilleke, Bojun Liu, Yue Wu, Michael S. O’Connor, Xuhui Huang

**Affiliations:** Department of Chemistry, Theoretical Chemistry Institute, 5228 University of Wisconsin-Madison, Madison, Wisconsin 53706, United States

## Abstract

Conformational changes essential for protein function involve transitions through multiple short-lived, high-energy states within the complex free energy landscape. While existing methods, such as Markov State Models and non-Markovian approaches built from molecular dynamics (MD) simulations, can effectively capture metastable states, they struggle to identify transition states. Transition state identification via dispersion and variational principle regularized neural networks (TS-DAR) is a computational framework that utilizes out-of-distribution detection to systematically identify all transition states involved in specific biomolecular conformational changes. TS-DAR leverages a deep learning model to map protein conformations from MD simulations onto a hyperspherical latent space. This low-dimensional representation retains the critical kinetic information of biomolecular conformational changes. To distinguish metastable states from transition states, TS-DAR utilizes a VAMP-2 and dispersion loss function, enabling the automated identification of transition state conformations. This framework provides a comprehensive view of protein conformational landscapes and facilitates studies on drug binding, enzyme activity, and mutation effects. This tutorial aims to guide researchers through the implementation and application of TS-DAR, highlighting its utility in computational biophysics. The code for this tutorial is available at: https://github.com/xuhuihuang/ts-dar-tutorials.

## Graphical abstract

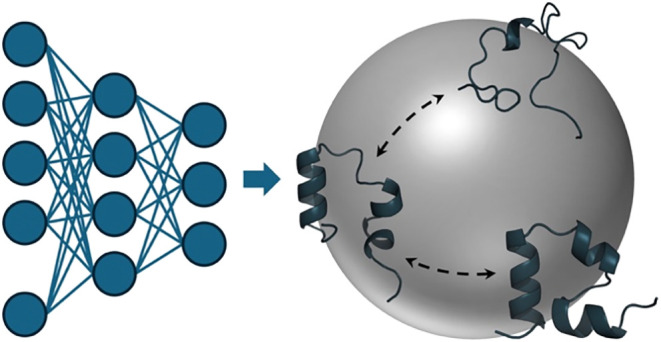

## 1 Introduction

Protein conformational changes are crucial for their biological function, driving processes such as enzyme catalysis, signal transduction, and allosteric regulation.^1,2^ Accurately modeling these molecular processes is essential for understanding biomolecular mechanisms and developing targeted therapeutics.^3^ Markov State Models (MSMs)^3–16^ and non-Markovian approaches,^17–20^ such as quasi-MSMs^17^ and the Integrative Generalized Master Equation (IGME)^20^ model, built from extensive molecular dynamics (MD) simulations, are powerful tools for identifying metastable states and characterizing their transitions. However, one persistent challenge in this field is the identification of transition states—critical, yet sparsely populated conformations that define the rate-limiting steps of molecular processes.^21^ A promising solution to this challenge arises from advances in out-of-distribution (OOD) detection, a concept originally developed to improve the reliability of artificial intelligence (AI) in high-risk applications such as self-driving cars.^22,23^ In this context, OOD detection ensures that autonomous vehicles do not make incorrect predictions when encountering unfamiliar scenarios, such as unusual road conditions or unexpected obstacles. Analogously, in protein dynamics, transition states occupy sparsely populated regions in the free energy landscape, making them inherently OOD relative to the well-characterized metastable states.^21^ By leveraging OOD detection, machine learning models can systematically identify these rare conformations, providing a comprehensive view of biomolecular conformational changes.

In this tutorial, we provide a guide to transition state identification via dispersion and variational principle regularized neural networks (TS-DAR),^21^ a deep learning framework that integrates OOD detection to accurately and simultaneously identify all transition states associated with biomolecular conformational changes. Within this framework, an encoder neural network maps protein conformations from MD simulations onto a hyperspherical latent space.^21^ This latent space provides a compressed, lower-dimensional representation of the protein’s conformational states while retaining the key kinetic information. Within this hyperspherical latent space, TS-DAR utilizes a dispersion loss function to enforce equal separation between metastable states, enabling the automated and systematic detection of all transition states. By analyzing the structural ensembles of these transition state conformations, users can directly probe the key chemical interactions that enable proteins to overcome free energy barriers.

Liu et al.^21^ demonstrated the efficacy of TS-DAR across multiple systems, ranging from simplified 2D potentials and alanine dipeptide to complex biomolecules such as the DNA motor protein AlkD. In the case of AlkD translocation on dsDNA, TS-DAR uncovered novel insights into the role of protein–DNA hydrogen bonds in the rate-limiting step of translocation. Across all tested systems, TS-DAR outperformed previous methods, including MaxEnt-VAMPNets^24^ and MSM-committor,^25^ in both accuracy and efficiency of transition state identification. By providing a robust framework for mapping the protein’s conformational landscape, TS-DAR enhances our ability to study the effects of mutations and drug binding. Insight into these key mechanistic details advances our understanding of enzyme function and disease progression, thereby facilitating more effective therapeutic design.

**Figure 1:**
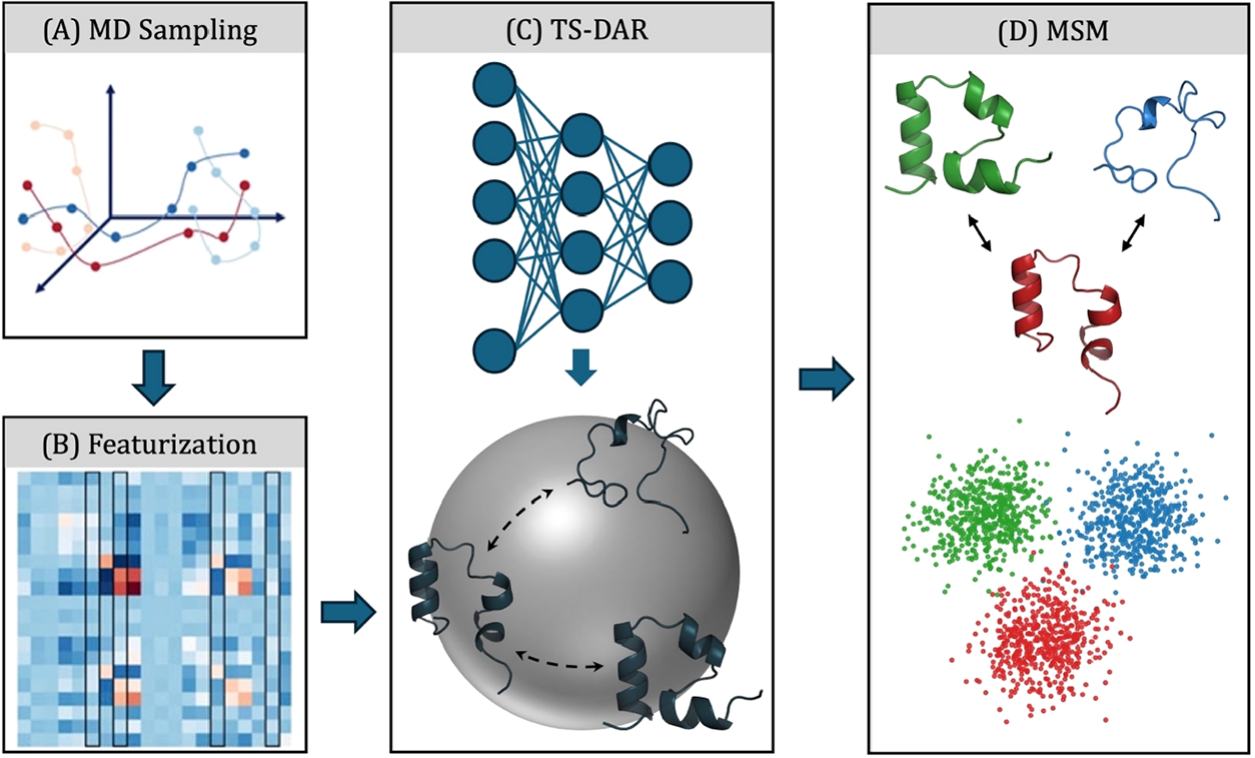
End-to-end pipeline for studying protein dynamics using TS-DAR. (A) Extensive MD simulations are performed between two or more functional conformational states. (B) Relevant features are selected to describe the system of interest. (C) TS-DAR uses a neural network to map molecular configurations onto a hyperspherical latent space. This latent space provides a compressed, lower-dimensional representation of the protein’s conformational states while retaining the key kinetic information. (D) The state assignments from TS-DAR can be used to construct a Markov State Model. Panels A and B are reproduced from ref 26 with permission from AIP Publishing.

## 2 TS-DAR Theory and Workflow for Protein Dynamics Analysis

In this section, we outline the conceptual framework behind TS-DAR and describe the workflow used to identify transition states from MD simulations. TS-DAR works by mapping MD-derived conformations onto a structured latent space, which enables the automated detection of transition states. The protocol consists of four main steps: MD sampling, featurization, TS-DAR modeling, and MSM construction (Figure 1).

### 2.1 Featurization

Once you have obtained MD simulation data for your system, the next step is to reduce its dimensionality by selecting structural features that capture the most relevant conformational dynamics. This can be done using automatic feature selection methods such as spectral accelerated sequential incoherent selection (spectral oASIS)^27^ or molecular systems automatic identification of correlation (MoSAIC).^27,28^ Based on the variational principle of conformational dynamics, spectral oASIS efficiently identifies a subset of features that best capture the slow dynamics of the system, eliminating the need to analyze the entire data set.^27^ This makes it a powerful tool for studying large-scale molecular simulations. MoSAIC, on the other hand, is a correlation-based feature selection method that uses the Leiden community detection algorithm to group similar features into clusters. These clusters are then ranked by size, where larger clusters are thought to represent collective motions and thus more meaningful dynamic processes, while smaller clusters are regarded as noise and ignored.^28^

**Figure 2:**
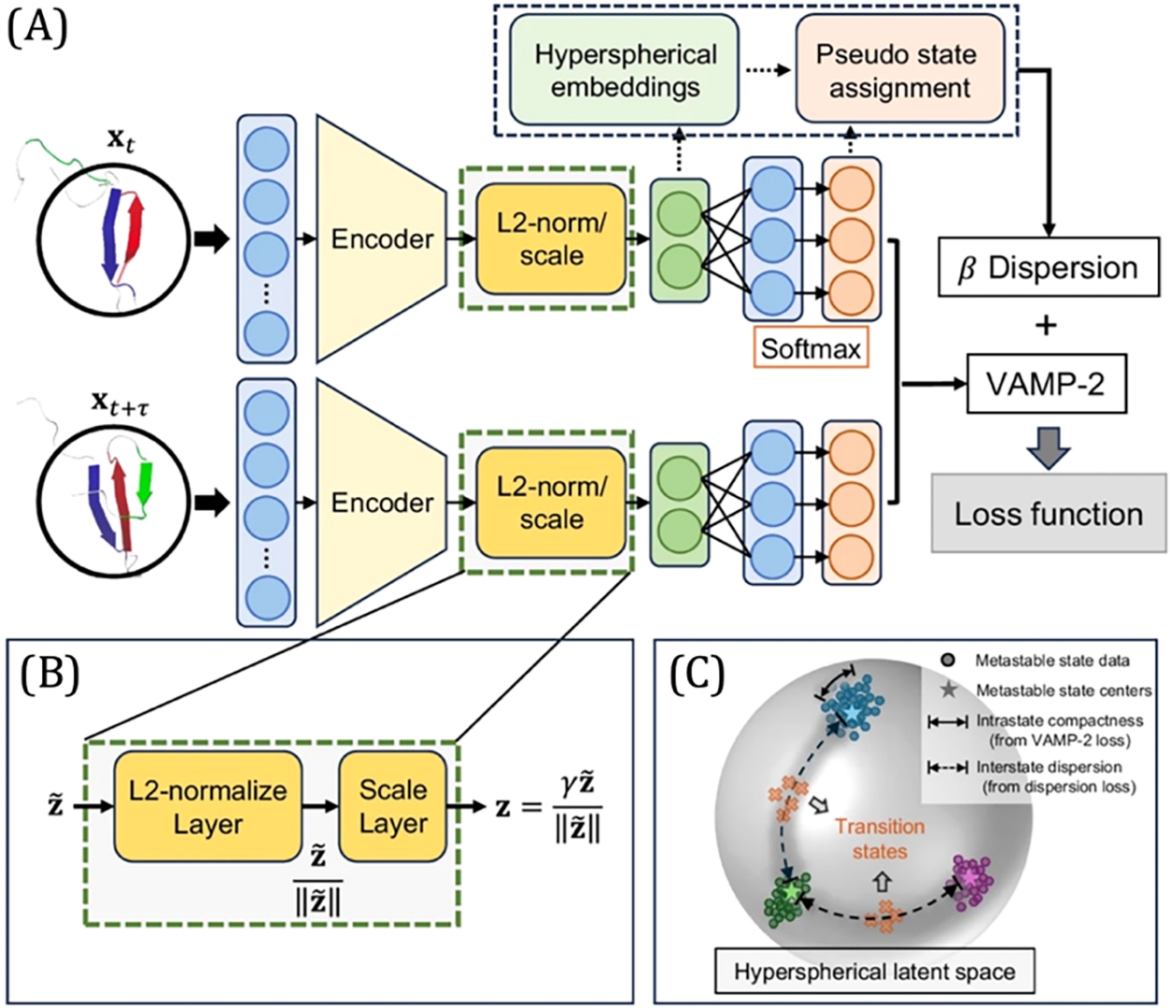
Overview of the TS-DAR framework. (A) TS-DAR uses transition pairs (*x*_*t*_ and *x*_*t*+τ_) from molecular dynamics trajectories as input. It includes an L2-norm layer to generate the hyperspherical embeddings. The Softmax outputs are used to obtain pseudostate assignments. The hyperspherical embeddings and pseudostate assignments are used to estimate the loss function. TS-DAR optimizes the neural network using a combined loss function, which includes the VAMP-2 loss and a weighted dispersion loss. (B) The L2-norm layer confines the feature embeddings (*z̃*) within a hypersphere of radius γ, resulting in the hyperspherical embeddings (*z*). (C) The hyperspherical latent space. The circles represent metastable state data; the stars denote the metastable state centers. The solid arrows highlight intrastate compactness (from the VAMP-2 loss), while the dashed arrows highlight interstate dispersion (from the dispersion loss). This figure is reproduced from ref 21, licensed under CC-BY-NC-ND 4.0.

### 2.2 TS-DAR Framework

The model architecture of TS-DAR consists of three parts (Figure 2A). The first part contains an encoder neural network responsible for processing the input features. TS-DAR uses transition pairs (*x*_*t*_ and *x*_*t*+τ_) from MD trajectories as input. The second part applies an L2-norm constraint to place all the feature embeddings (z̃) on a fixed-radius hypersphere. This step is divided into two stages: first, the feature vectors at the penultimate layer (i.e., the last hidden layer or the second-to-last layer of a neural network) are divided by their L2-norms; then, they are rescaled by a scaling factor to confine z̃ within the hypersphere (Figure 2B). The third part of the model is a linear transformation layer that maps the hyperspherical latent features onto a set of output nodes. This is followed by a Softmax function that converts these outputs into pseudostate assignment probabilities. These assignments, along with the hyperspherical embeddings, are then used to compute the model’s loss, which has two components: the VAMP-2 loss and the dispersion loss.^21,29^

#### 2.2.1 Loss Functions

To accurately and simultaneously identify transition states and ensure that the learned latent space is well-dispersed, TS-DAR leverages a combination of loss functions. A loss function is a scalar objective that the model tries to minimize during training (e.g., mean squared error or cross-entropy). It is a tool that quantifies the error between model predictions and true values or target values. TS-DAR specifically employs the VAMP-2 loss and dispersion loss.^21,29^ The VAMP-2 loss is derived from the Variational Approach for Markov Processes (VAMP), which aims to extract the slow collective variables from the data.^29^ Given an MD trajectory of length *T*, a batch of transition pairs {(*x*_*t*_,*x*_*t*+τ_)}_*t*=1_^*b*^ with lag time, τ, is sampled. This produces two batches of data: **B** = [*x*_1_,*x*_2_, ···, *x*_*b*_]^*T*^ and **B̂** = [*x*_1+τ_,*x*_2+τ_, ···, *x*_*b*+τ_]^*T*^, which are fed into two parallel network lobes with shared parameters. Each lobe outputs Softmax-transformed basis functions (χ) applied to the input features, yielding: **X** = [χ­(*x*_1_), ···, χ­(*x*_*b*_)]^*T*^ and **Y** = [χ­(*x*_1+τ_), ···, χ­(*x*_*b*+τ_)]^*T*^. The remove-mean time-instantaneous (**C̅**_00_ and **C̅**_11_) and time-lagged (**C̅**_01_) correlation matrices can then be calculated as follows

1 $$\begin{array}{l} {\mathrm{C̅}}_{00}=\frac{1}{T-\tau }X^{T}X-\pi_{0}\pi_{0}^{T}\\ {\mathrm{C̅}}_{11}=\frac{1}{T-\tau }Y^{T}Y-\pi_{1}\pi_{1}^{T}\\ {\mathrm{C̅}}_{01}=\frac{1}{T-\tau }X^{T}Y-\pi_{0}\pi_{1}^{T} \end{array}$$

where **π**_**0**_ and **π**_**1**_ are mean vectors of **X** and **Y**, respectively, given by: $\pi_{0}=\frac{1}{T-\tau }X^{T}1$ and $\pi_{1}=\frac{1}{T-\tau }Y^{T}1$ . These correlation matrices are then used to calculate the VAMP-2 loss, which is defined as

2 $$L_{\mathrm{vamp}2}=-{∥{{\mathrm{C̅}}_{00}}^{-\left(1/2\right)}{\mathrm{C̅}}_{01}{{\mathrm{C̅}}_{11}}^{-(1/2)}∥}_{F}^{2}-1$$

Minimizing the VAMP-2 loss ensures that the hyperspherical embeddings of the MD conformations are arranged according to their kinetic metastability, effectively capturing the system’s slowest dynamics (Figure 2C). While the VAMP-2 loss promotes intrastate compactness, it can lead to a latent space where different metastable states are unevenly distributed. To address this, TS-DAR incorporates a dispersion loss that regularizes the hyperspherical latent space, ensuring that the metastable state centers (i.e., free energy minima) are uniformly distributed across the hypersphere. As a result, all transition state conformations, located between free energy basins, can be automatically and simultaneously identified based on their cosine similarities to the state centers. The dispersion loss is defined as follows

3 $$L_{\mathrm{dis}}=\frac{1}{C}\sum_{i=1}^{C}\log\frac{1}{C-1}\sum_{j=1}^{C}1\left\{j\neq i\right\}e^{\mu_{i}^{T}\mu_{j}/\sigma }$$

where *C* corresponds to the number of states, σ is a scaling hyperparameter, and **μ**_***i***_ and **μ**_***j***_ represent the state center vectors for states *i* and *j*, respectively. Note that this computation is time-consuming, as it utilizes a moving average algorithm that iterates through all conformations in each state to calculate the state center vectors. Overall, the TS-DAR framework uses a weighted combination of the VAMP-2 loss and dispersion loss, given by

4 $$L_{\mathrm{total}}=L_{\mathrm{vamp}2}+{\beta L}_{\mathrm{dis}}$$

To learn the latent space representation of the protein conformations, we extract selected structural features (e.g., atomic coordinates, pairwise distances, or torsion angles) from the simulation data. Then, the encoder in the TS-DAR framework learns to compress these features into a lower-dimensional latent space. During training, the model learns the distribution of conformational states, allowing it to recognize transition state conformations within the latent space. To evaluate the TS-DAR model, plot the VAMP-2 loss vs. the number of training epochs. The VAMP-2 loss measures how well the learned latent space captures the slowest dynamic modes. Training should continue until the VAMP-2 loss converges, indicating that the loss function has been optimized, and the model has effectively learned patterns from the data (see Section 3 for examples). If training is stopped prematurely, the model might fail to learn meaningful patterns. Insufficient training results in underfitting, with high errors on both training and validation sets. Conversely, excessive training can lead to overfitting, where the model memorizes the training data but performs poorly on new data.

#### 2.2.2 Hyperparameters

When training the model, it is important to keep hyperparameters in mind. Hyperparameters are tunable settings chosen before training to control how the model learns. A few important hyperparameters to consider are listed below

##### 2.2.2.1 Batch Size

The number of samples processed in one complete propagation. During the forward propagation, the model computes predictions and loss for a subset of data (batch); during the backward propagation, the model calculates the gradient based on the loss to update its parameters. This gradient is an estimate of the true gradient (which is computed over the entire data set), and its accuracy improves with larger batch sizes. In TS-DAR, smaller batches yield noisier gradients, which may improve generalization and reduce memory usage but can increase training time if the learning rate is not adjusted. Conversely, larger batches yield smoother gradients but may lead to sharper minima with poorer generalization. Therefore, the optimal batch size should be determined by monitoring both training and validation performance.

##### 2.2.2.2 Network Architecture

TS-DAR uses an encoder neural network to map molecular configurations to a low-dimensional latent representation. This network consists of a multilayer perceptron (MLP) with several hidden layers. For example, in one configuration used for alanine dipeptide, the encoder network takes an input feature vector (e.g., *x*, *y*, *z* coordinates) and passes it through hidden layers of specified sizes (e.g., 30, 30, 30, 30, 10 neurons) before outputting a latent vector of dimension feat_dim (e.g., 2). This example architecture 30–30–30–30–10–2 represents an input layer with 30 features, four hidden layers with 30, 30, 30, and 10 neurons, respectively, and an output layer with 2 units (the latent features). Notably, TS-DAR uses hyperspherical latent embeddings, so after the penultimate layer, the latent vector is L2-normalized to lie on a unit hypersphere. This ensures that the model focuses on angular separation in the latent space, which is important for distinguishing metastable states from transition states.

##### 2.2.2.3 Learning Rate

Controls the step size used during each weight update, directly influencing how quickly the model learns. If set too high, training can become unstable, causing the loss to oscillate or diverge. If set too low, convergence becomes slow and may stall altogether. A typical starting value, such as 0.001, often balances speed and stability, but the optimal value should be determined by monitoring both training and validation losses. Adjustments via learning rate schedules or grid searches can help identify the optimal value to ensure steady, reliable convergence.

##### 2.2.2.4 β

The weight for the dispersion loss relative to the VAMP-2 loss. It determines how strongly the model penalizes large cluster radii in the latent space, encouraging tighter and more well-separated clusters. The value of β has been empirically optimized to 0.01, which has been shown to generalize well across different data sets.

##### 2.2.2.5 Feat_dim

The number of dimensions in the model’s latent space. For example, feat_dim = 2 means the embeddings lie on a circle. We recommend using feat_dim = 2 for models with three or fewer states, and feat_dim = 3 for models with four or more states.

##### 2.2.2.6 N_states

Defines the number of metastable states the model expects, or equivalently, the number of clusters with low OOD scores that it forms in the latent space. This parameter guides clustering: setting too few clusters may merge distinct states, while too many clusters can artificially split a single state.

##### 2.2.2.7 Pretrain

An initial training phase (e.g., 10 epochs) before the main training, during which the network learns a basic latent representation, typically without the dispersion regularization. This phase, similar to VAMPnet, helps the network roughly separate metastable states, providing a solid foundation for subsequent refinement with full regularization. Pretraining prevents issues such as collapsing or the arbitrary splitting of data that can occur if complex loss functions are applied from the start. Overall, pretraining acts as a warm-up, leading to faster convergence and a more stable final performance.

##### 2.2.2.8 N_epochs

The total number of passes over the training set, including the pretraining phase. After each epoch, the model updates its weights based on all training examples (processed in batches). Too few epochs can cause underfitting, while too many may lead to overfitting. Monitoring validation performance, ideally with early stopping, helps in selecting an appropriate number of epochs. In practice, for a simple system like alanine dipeptide, a few dozen epochs are often sufficient for convergence.

#### 2.2.3 Computing OOD Scores and Identifying Transition States

After training the TS-DAR model, we can identify potential transition state structures in the latent space by computing the OOD scores (eq 5). Since TS-DAR is trained on the time-lagged correlations of input features, we expect that the hyperspherical latent space preserves the kinetic geometry of the system. Therefore, MD conformations with high OOD scores that lie between metastable states on the hypersphere likely represent transition state structures. Within the hyperspherical latent space, metastable states are represented by state center vectors, which are optimized using the dispersion loss to ensure an even distribution across the hypersphere. Once these centers are established, the OOD score for each conformation is obtained by taking the cosine similarity between its feature vector and the metastable state center vectors.

5 $$\mathrm{OOD}\;\mathrm{score}=-\max\left\{z^{T}{\left\{\mu_{c}\right\}}_{c=1}^{C}\right\}+1$$

where ***z*** represents the hyperspherical latent embedding of a conformation, and **μ**_***c***_ represents the state center vector for states *c*. The resulting angular distance quantifies how much a conformation deviates from the nearest metastable state, with larger angles indicating higher OOD scores. After computing the OOD scores, one will be able to visualize the hyperspherical latent space, which reveals clusters of metastable conformations and outliers (see Section 3 for examples). To identify potential transition state structures, biomolecular conformations with OOD scores above a chosen threshold are selected. There are two approaches to setting the threshold. By default

6 $$\mathrm{thres}=0.5\times \left(-\cos(\frac{\theta^{*}}{2})+1\right)$$

where θ* represents the angle between two nearest-neighbor state-center vectors on the hypersphere. Alternatively, users can define the OOD threshold to yield a desired number of transition state candidates by setting the threshold based on the observed distribution of OOD scores. This approach is typically used when some prior intuition about the data set is available; otherwise, we recommend using the default threshold. In our previous work, we show that transition state conformations identified with high TS-DAR OOD scores correlate with a committor value of 0.5 for simple systems.^21^ This indicates an equal probability of proceeding to either the product or reactant state, which is a defining characteristic of a transition state. Nevertheless, we note that TS-DAR provides candidate transition state structures, and we encourage users to establish their validity via committor analysis.

**Figure 3:**
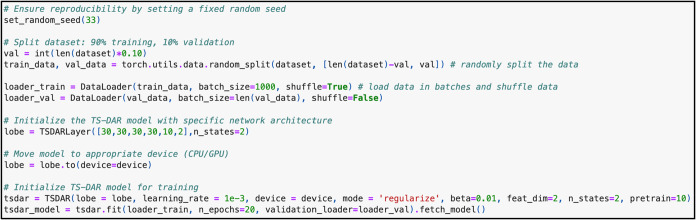
Code required to train a TS-DAR model for alanine dipeptide.

**Figure 4:**
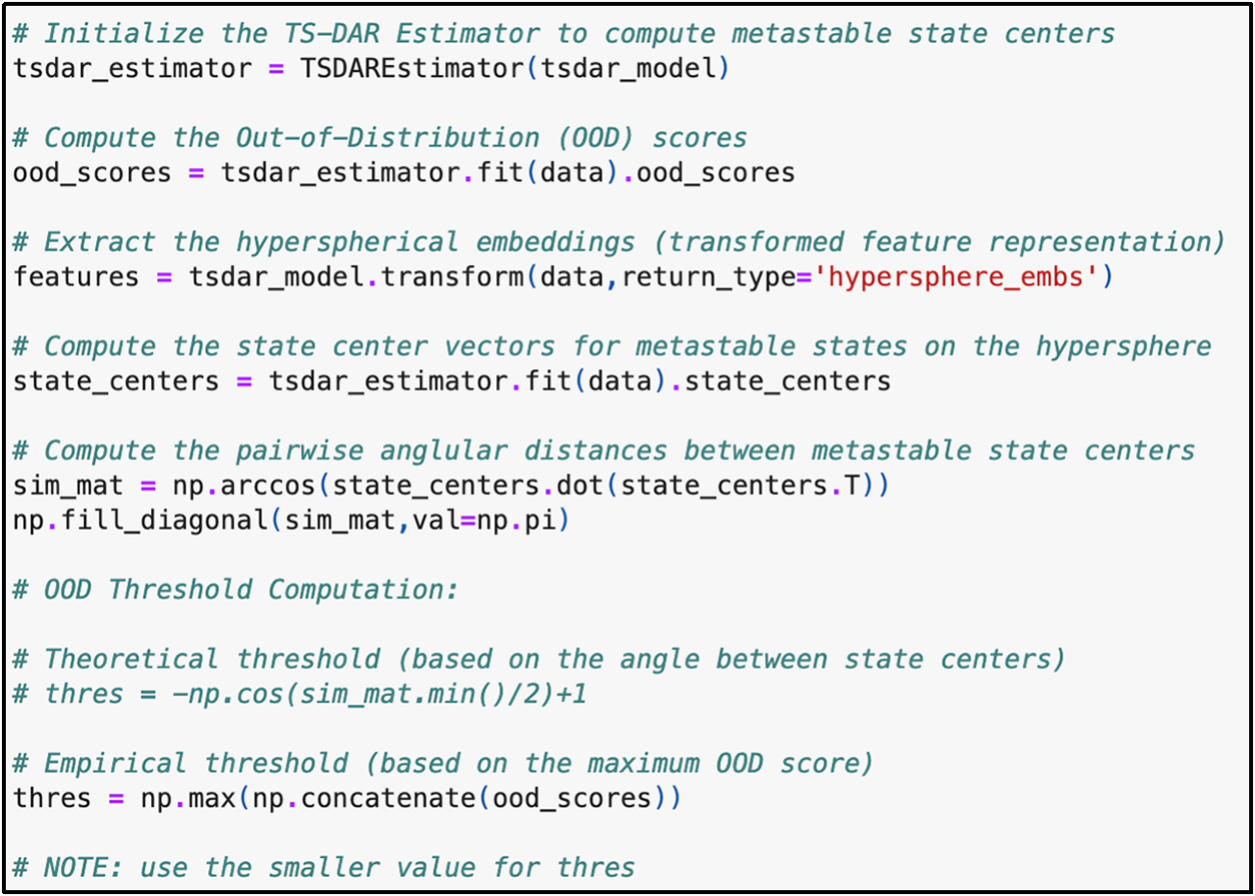
Code used to estimate the metastable state centers and compute the OOD scores.

### 2.3 Kinetic Modeling Using TS-DAR-Derived States

After the TS-DAR model is trained and the metastable and transition states are identified within the hyperspherical latent space, users can construct an MSM using the TS-DAR state assignments. MSMs provide a well-established framework for quantitatively describing the kinetics of biomolecular systems by modeling the transitions between discrete conformational states as Markov processes.^3–16^ Using the TS-DAR-derived state discretization, the MSM estimates transition probabilities between states over a chosen lag time. This enables calculation of key kinetic properties such as transition rates, relaxation timescales, and mean first passage times. The accuracy of the MSM critically depends on the quality of the underlying state definitions. To validate the MSM, you can perform a Chapman–Kolmogorov (CK) test. The CK test compares the predicted evolution of state probabilities (calculated via successive powers of the transition matrix) with those directly observed from the MD trajectories. A close match between the TS-DAR-based MSM predictions and the MD data confirms that the MSM accurately captures the essential long-time scale kinetics of the system. This integration of TS-DAR and MSM enables accurate identification of rare conformational states along with robust kinetic modeling, providing a powerful framework for analyzing protein conformational dynamics.

## 3 Tutorial Examples

In this section, we demonstrate how to apply the TS-DAR framework to analyze MD data for three different data sets: alanine dipeptide, the villin headpiece (named “HP35”), and protein phosphatase 2A (PP2A). The first step is to download and install Anaconda and use it to create a new environment. Then, install the TS-DAR source code locally. The source code for TS-DAR is available at https://github.com/xuhuihuang/ts-dar. The example code for this tutorial is available at https://github.com/xuhuihuang/ts-dar-tutorials.

### 3.1 Alanine Dipeptide

First, we apply the TS-DAR framework to analyze MD data for alanine dipeptide. The data set consists of three 250 ns trajectories, with a saving interval of 1 ps (resulting in a total of 750,000 molecular conformations). The conformations were aligned to the first frame. Before using TS-DAR, the MD trajectories must be preprocessed to generate input data sets, where the lag time is specified (1 ps in this example). Since alanine dipeptide is a small system, feature selection is unnecessary. In this example, the x, y, and z coordinates of the 10 heavy atoms (30 features total) are used as input to the model. The encoder in the framework will learn to compress these input features into a lower-dimensional latent space.

To train the model, there are several hyperparameters to keep in mind (Figure 3). First, you can set the random seed using set_random_seed­(*x*), where *x* is an integer used to initialize the pseudorandom number generators in the training pipeline (e.g., for data shuffling). By setting the same seed for every run, all “random” operations produce the same sequence of outcomes, making training results reproducible. Without a fixed seed, each training run could lead to slightly different results, making it hard to trace issues or reliably benchmark the model’s performance. Next, choose the percentage of trajectories to use for training versus validation. A 90/10 split is common, as it provides sufficient data for both model optimization and performance evaluation, especially when the data set is large, where 10% still represents a meaningful subset. Once all the hyperparameters are set, you can start training. For reference, it takes approximately 5.5 min to train a TS-DAR model for alanine dipeptide on an Apple M3 Mac (20 epochs total). It takes around 30 s to pretrain (first 10 epochs) and 5 min to complete training (last 10 epochs).

To evaluate the TS-DAR model, plot the loss vs. the number of training epochs using plt.plot­(tsdart.validation_vamp) and plt.plot­(tsdart.validation_dis) to assess convergence (Figure 5B,C). Once the model has converged, it can be used to estimate the metastable state centers and compute the OOD scores (Figure 4). These OOD scores, which quantify the likelihood of a conformation being in a transition state, can be projected onto the free energy landscape of alanine dipeptide (Figure 5D,E). Additionally, projection onto the hypersphere aids in visualizing both metastable states and transition states (Figure 5F).

**Figure 5:**
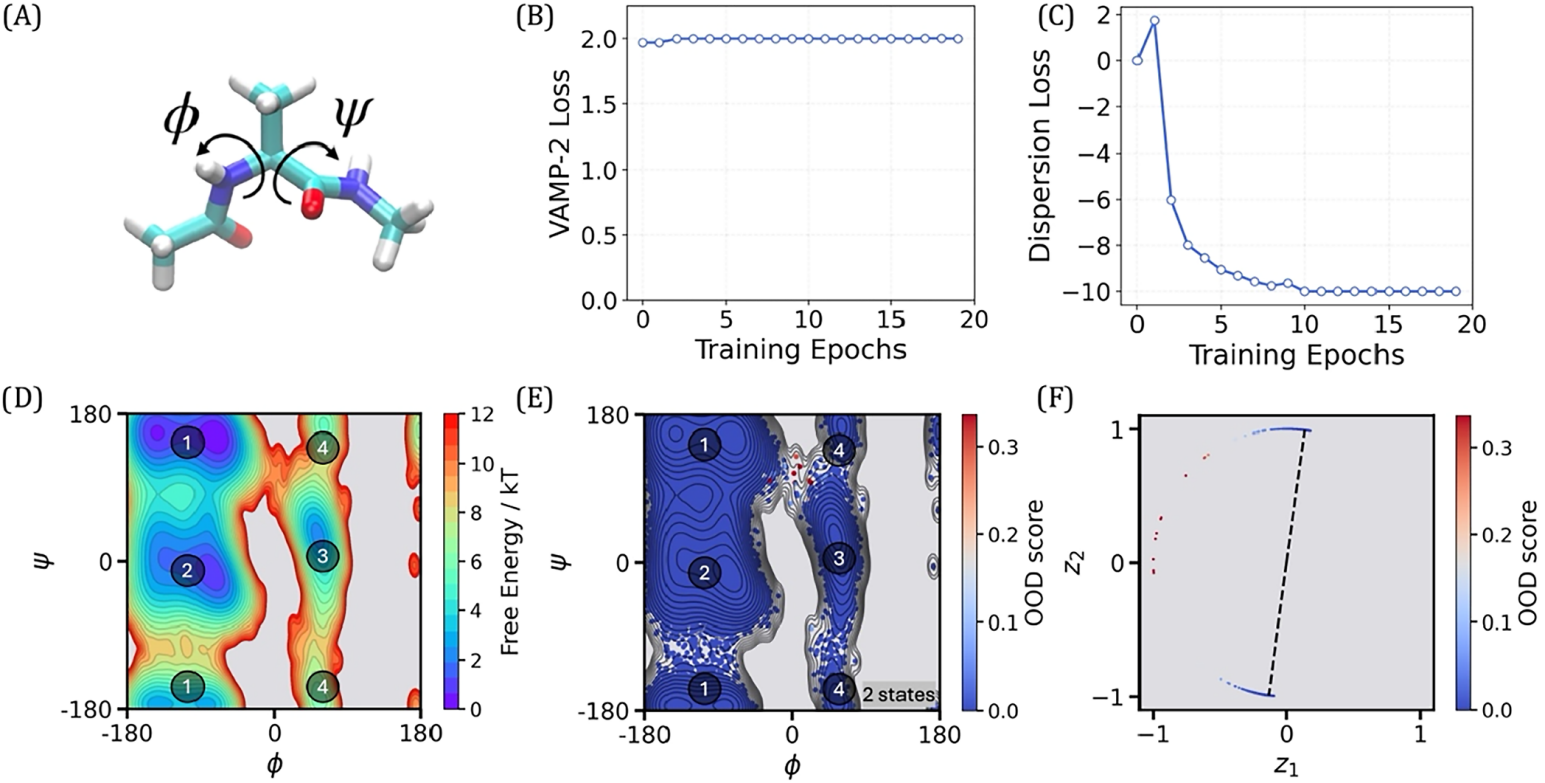
2-state TS-DAR model for alanine dipeptide. (A) Alanine dipeptide molecule with the backbone torsion angles, ϕ and ψ, labeled. (B) Validation curve for the VAMP-2 loss. (C) Validation curve for the dispersion loss. (D) Free energy landscape projected onto the two backbone torsion angles, ϕ and ψ. The four basins are labeled in gray. State 1 corresponds to basins 1 and 2, and state 2 corresponds to basins 3 and 4. (E) OOD scores projected onto the two backbone torsion angles, ϕ and ψ. Conformations with high OOD scores are shown in red. (F) Hyperspherical latent representation of molecular conformations according to OOD scores. The dashed lines point to the metastable state centers.

This example demonstrates a 2-state TS-DAR model, where state 1 corresponds to basins 1 and 2, and state 2 corresponds to basins 3 and 4, as shown in Figure 5D. As you go through the Jupyter notebook for the alanine dipeptide system, you will be able to use the steps outlined above to build a 3-state and 4-state model (Figure 6). It is important to note that the number of metastable states for a system should be chosen a priori. The recommended approach is to perform a time-lagged independent component analysis (tICA)^30,31^ and then examine the implied timescales (ITS) plot. The ITS plot shows the dominant relaxation timescales as a function of lag time, helping to confirm that the dynamics are Markovian and that the model is robust.^32^ The optimal number of metastable states can then be determined based on the spectral gaps in the ITS plot (i.e., by identifying the dynamical modes that are clearly separated from the faster modes), where the number of metastable states is taken as one plus the count of well-separated slow timescales. We note that tICA is used solely for estimating the number of states, and that TS-DAR training is independent of tICA. In the next example on the villin headpiece, we follow the protocol published by Wu et al. to determine the number of metastable states.^26^ For more details on how to build a microstate MSM and use the ITS to determine the number of metastable states before using TS-DAR, see ref 26.

**Figure 6:**
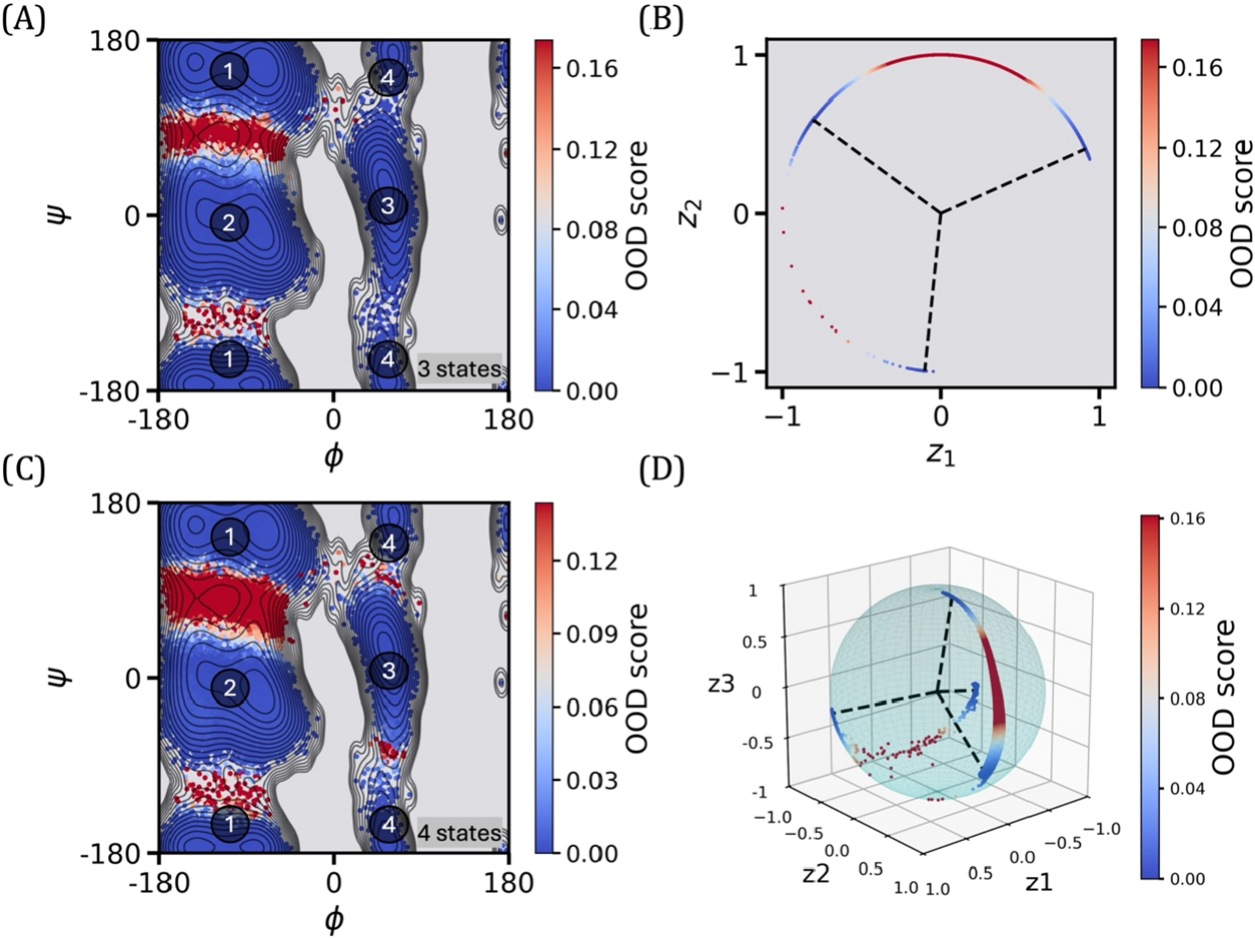
3-state and 4-state TS-DAR model for alanine dipeptide. (A) OOD scores projected onto the two backbone torsion angles, ϕ and ψ, for the 3-state model. In this model, state 1 corresponds to basin 1, state 2 corresponds to basin 2, and state 3 corresponds to basins 3 and 4. (B) Hyperspherical latent representation of molecular conformations according to OOD scores obtained from the 3-state TS-DAR model. The dashed lines point to the metastable state centers. (C) OOD scores projected onto the two backbone torsion angles, ϕ and ψ, for the 4-state model. In this model, states 1 through 4 correspond to basins 1 through 4, respectively. (D) Hyperspherical latent representation of molecular conformations according to OOD scores obtained from the 4-state TS-DAR model. The dashed lines point to the metastable state centers.

### 3.2 Villin Headpiece (HP35)

For the second example, we apply the TS-DAR framework to analyze MD data for the villin headpiece system (HP35). The data set is from D.E. Shaw Research^33^ and consists of a single 300 μs all-atom trajectory of the Nle/Nle mutant of HP35 (PDB ID: 2F4K), with a saving interval of 200 ps. Since HP35 is a larger system, we need to perform featurization and build a microstate MSM to determine the appropriate number of macrostates to use to build a TS-DAR model. For this tutorial, we follow the protocol in ref 26. We recommend reviewing that tutorial for more background on MSM construction and state selection.

Following the tutorial by Wu et al., we use the 528 pairwise distances between C-α atoms (with a separation of at least three residues) as the raw input features for model training. For reference, it takes approximately 20 min to train a TS-DAR model for HP35 on an Apple M3 Mac (30 epochs total). It takes around 3 min to pretrain (first 15 epochs) and 17 min to complete training (last 15 epochs). To evaluate the TS-DAR model, plot the loss vs. the number of training epochs using plt.plot­(tsdart.validation_vamp) and plt.plot­(tsdart.validation_dis) to assess convergence (Figure 7A,B). Once the model has converged, it can be used to estimate the metastable state centers and compute the OOD scores. Most of the code is similar to that of the alanine dipeptide example.

**Figure 7:**
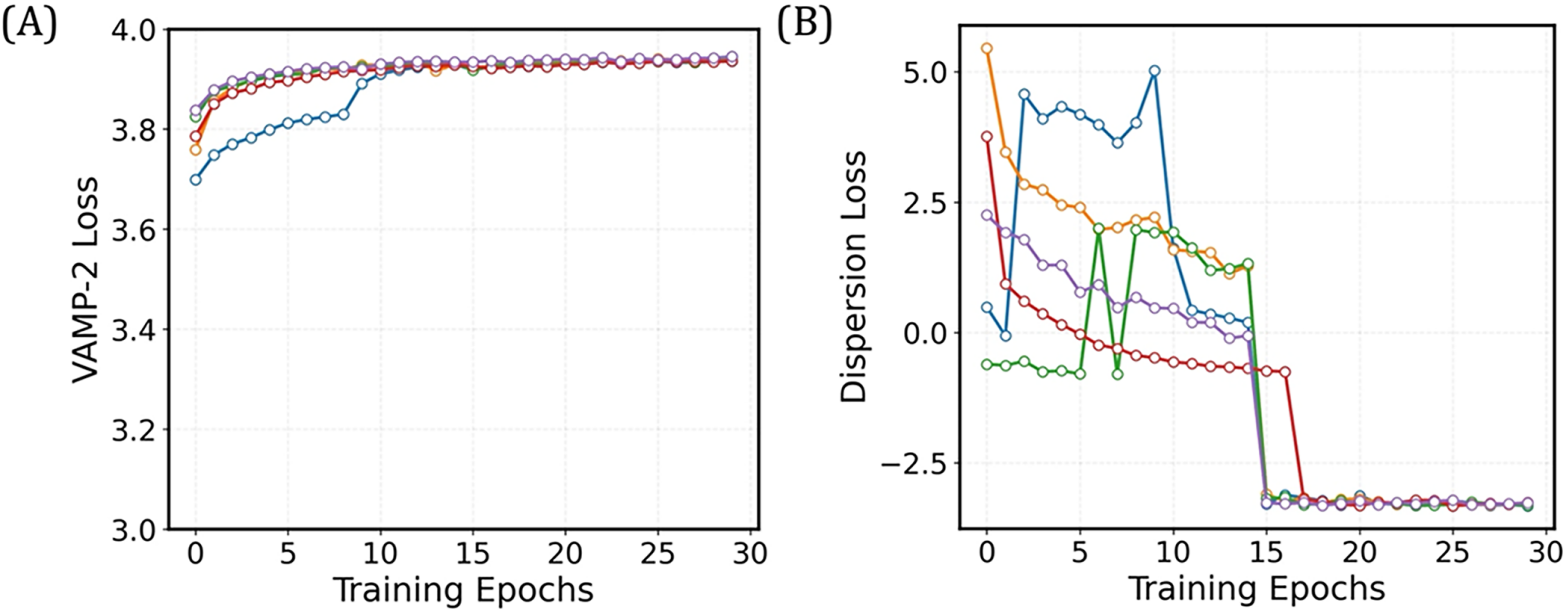
Validation curves for the 4-State TS-DAR model for villin headpiece. (A) Validation curve for the VAMP-2 loss. (B) Validation curve for the dispersion loss.

In this example, we include additional code to describe the StateAnalyzer function. This function identifies frames from the MD data that are assigned to state centers (metastable states) as well as the frames with high OOD scores (transition states). The code below identifies the frames assigned to a specified state center (Figure 8). Currently, center_idx is set to 0, which provides the frame indices for state 1. Changing the value of center_idx will return the frame indices that correspond to other states.

**Figure 8:**
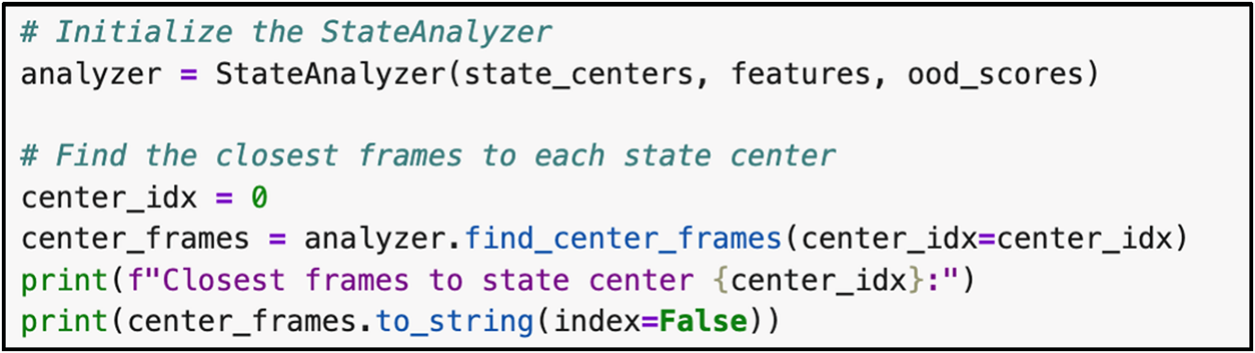
StateAnalyzer function for identifying frames assigned to metastable states.

The next part of the code is used to identify frames assigned to possible transition states (Figure 9). The code below identifies the simulation frames between state 1 and state 2 that have high OOD scores. Changing the values of state_idx_1 and state_idx_2 will allow you to obtain the frame index for the transition states between different states.

**Figure 9:**
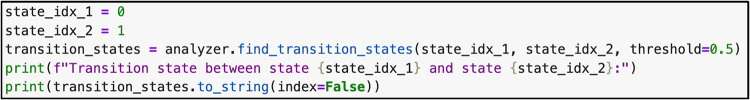
StateAnalyzer function for identifying frames assigned to possible transition states.

Once you obtain the frame indices for the metastable states and the transition states, you can output those frames as a .pdb file (Figure 10B–G). It is important to note that you should visualize several frames for each metastable state to get an idea of the protein conformation for that specific state; picking just one frame could lead to errors in the protein conformation, as it could represent a conformation slightly outside the state of interest. For transition states, picking the frames with the highest-scoring OOD scores is sufficient. To verify that the identified transition states are proper, users can run a committor analysis.

Once you output the conformations of interest, you can use them for further investigation. For example, you might assess how point mutations or ligand binding influence the stability of interaction networks, alter allosteric communication pathways, or shift the protein’s conformational ensemble. These investigations provide valuable insight into the molecular mechanisms underlying function and regulation and can identify potential therapeutic targets.

Additionally, by using the TS-DAR-derived state assignments, you can construct an MSM to describe the system’s kinetics (Figure 11). To evaluate whether the MSM accurately reproduces the long-time scale behavior from the short-time scale transition probabilities, perform a CK test. The resulting plots show the residence probability of each macrostate as a function of lag time (Figure 12A). The CK test demonstrates that the transition probabilities derived from the TS-DAR-based MSM closely match the long-time dynamics seen in the MD data, demonstrating that the model captures the essential kinetic features of the system. In addition, the ITS plot shows the dominant relaxation timescales as a function of lag time, confirming that the dynamics are Markovian and that the model is robust (Figure 12B).

### 3.3 Protein Phosphatase 2A (PP2A)

For the third and final example, we apply the TS-DAR framework to analyze MD data for protein phosphatase 2A (PP2A), a key serine/threonine phosphatase involved in many cellular processes.^34–36^ Mutations in its B56δ regulatory subunit have been linked to intellectual disability and cancer, potentially due to the disruption of the holoenzyme’s inactive state, which is maintained by autoinhibition and relieved by phosphorylation-induced activation.^37–39^ In the inactive, wild-type holoenzyme, the N- and C-arms of B56δ block the active site and substrate binding, resulting in dual autoinhibition.^40^ Activation is thought to involve the release of the N- and C-arms from the holoenzyme core.^41^ However, the effects of disease-related mutations on this mechanism are not well understood. Since these mutations are located far from the active site, they likely act through allosteric pathways. A prior study using unbiased simulations could not capture the full activation mechanism but, instead, successfully mapped the allosteric network surrounding the active site.^41^ Using the MD simulation data set from their work, we show that TS-DAR can distinguish subtle conformational differences between the open and closed states of the active site.

The MD data for this system consists of ten 100 ns all-atom trajectories with a saving interval of 10 ps.^41^ For the raw input features, we use the 26,565 pairwise distances between the regulatory subunit B56δ (residues 61–90, 180–312, and 560–601 in Chain B) and the catalytic subunit (residues 55–60, 84–92, and 260–270 in Chain C). These features are then processed using spectral oASIS to identify those that best capture the system’s slowest dynamics. This results in a reduced set of 1000 features from the original 26,565. To assess whether this subset adequately describes the system’s kinetics, we plot the ITS using the 1,000 features. We then train ten TS-DAR models using these 1,000 features as input (Figure 13). For reference, it takes approximately 4 min to train a TS-DAR model for PP2A on an Apple M3 Mac (60 epochs total). It takes around 2.5 min to pretrain (first 50 epochs) and 1.5 min to complete training (last 10 epochs). To evaluate the TS-DAR model, plot the loss vs. the number of training epochs using plt.plot­(tsdart.validation_vamp) and plt.plot­(tsdart.validation_dis) to assess convergence (Figure 14A,B).

After training the model and ensuring it has converged, we extracted the active site opening distances, defined as the distance between the center of mass of residues 572–574 on the C-arm and the Mg^2+^ ions (Figure 15A), for each metastable state across all trajectories. We then computed kernel density estimates to obtain the average probability density distribution (Figure 15B). The resulting plot shows that state 2 samples more of the open active site conformation compared to state 1. State 1 predominantly samples the closed active site conformation, which has an active site opening distance of ∼0.85 nm (measured using PDB ID: 8U1X^40^). As a result, state 2 corresponds more to the open metastable state; however, it is important to note that the active site is not fully open (∼1.2 nm) due to limited sampling. Nevertheless, these results demonstrate that TS-DAR can effectively capture not only large-scale conformational changes but also subtle structural variations that are otherwise difficult to identify. This example highlights the versatility of TS-DAR in capturing a range of conformational changes in biomolecules.

**Figure 10:**
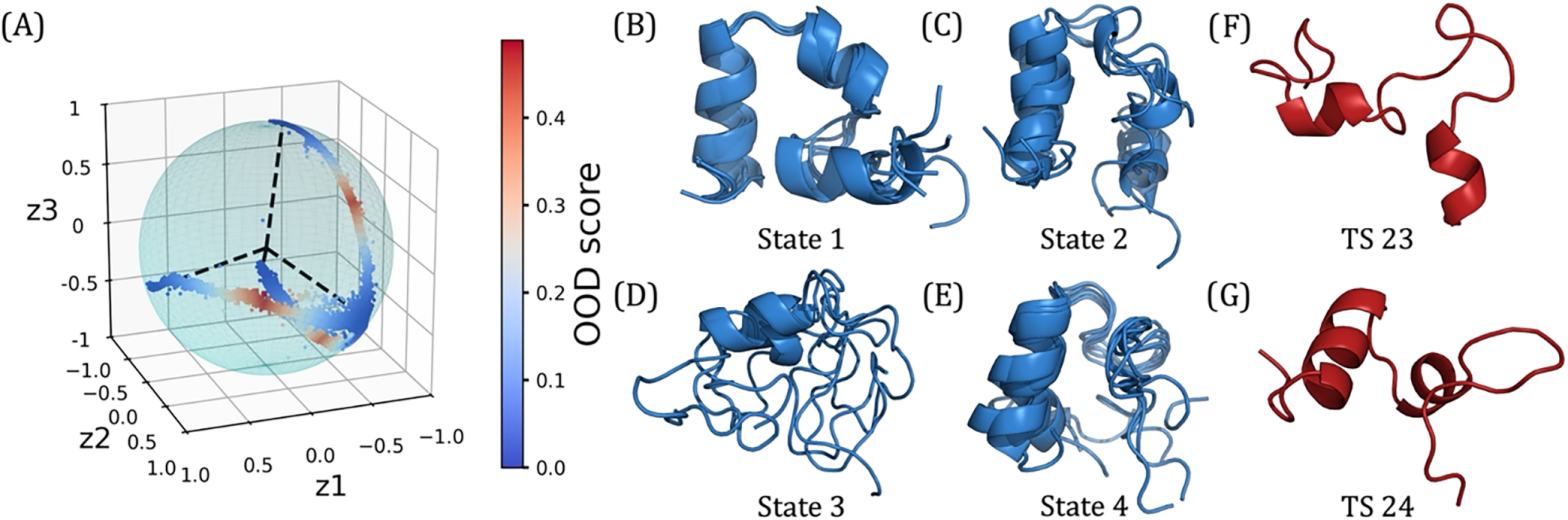
4-state TS-DAR model for villin headpiece with representative conformations for each metastable state and transition state. (A) Hyperspherical latent representation of molecular conformations according to OOD scores obtained from the 4-state TS-DAR model. The dashed lines point to the metastable state centers. (B–E) Overlay of five representative conformations from the TS-DAR model for metastable states 1 through 4. (F) Transition state conformation between states 2 and 3. (G) Transition state conformation between states 2 and 4.

**Figure 11:**
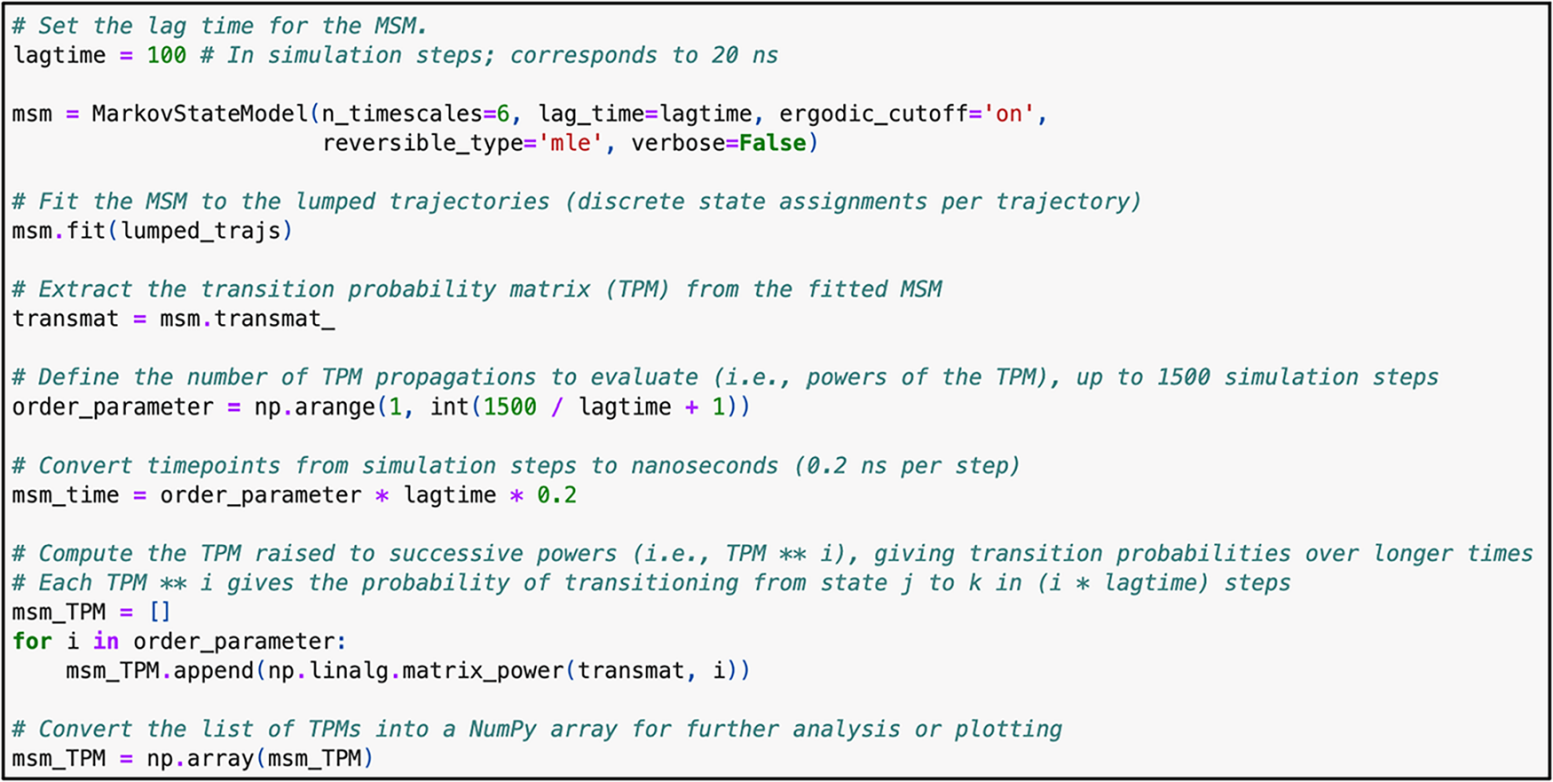
Code to construct a Markov State Model using the TS-DAR-derived state assignments.

## 4 Discussion and Future Perspective

In this tutorial, we provide a step-by-step guide to identify transition states from MD simulation data sets using TS-DAR. TS-DAR leverages a deep learning model to map protein conformations from MD simulations onto a structured, hyperspherical latent space. This latent space allows for a compressed, lower-dimensional representation of the protein’s conformational states while preserving the system’s essential kinetic behavior. Within this hyperspherical latent space, TS-DAR utilizes a combined VAMP-2 and dispersion loss function. The VAMP-2 loss ensures that conformations belonging to the same metastable state are allocated to a single basin, while the dispersion loss function enforces equal separation between metastable state centers in the hyperspherical latent space. This allows for the automated detection of transition state conformations.

While this tutorial focuses on applying TS-DAR to study alanine dipeptide, HP35, and PP2A, it is important to emphasize the method’s broader applicability. For example, transition state conformations identified by TS-DAR can serve as starting points for MD simulations to investigate how different antibiotics influence state populations during bacterial RNAP transcription. This approach enables researchers to systematically compare the effects of antibiotics during different stages of the transcription cycle (e.g., nucleotide addition, pausing, or translocation) and assess how each compound perturbs the conformational landscape. This strategy would provide mechanistic insight into how various inhibitors act and can inform the rational design or selection of antibiotics that most effectively disrupt key transition states in the bacterial transcription cycle.^42,43^ Another interesting application of TS-DAR is the analysis of noncanonical and metastable protein–protein interactions (PPIs), such as those formed in PROTAC-mediated ternary complexes. A key challenge in PROTAC design is identifying and stabilizing favorable encounter complexes (transient, loosely bound intermediates that form when a protein of interest and an E3 ligase come into proximity) through linker optimization that promotes productive ternary complex formation.^44^ TS-DAR can be applied to MD simulations of linker-free encounter complexes to identify metastable interface conformations that lie between the unbound and bound states. These conformations, marked by high OOD scores, represent kinetically relevant intermediates that can inform rational linker design. By revealing transient and functionally important protein–protein interfaces, TS-DAR provides a powerful and generalizable framework for advancing PPI-targeted drug discovery, particularly for next-generation degrader strategies such as PROTACs.

**Figure 12:**
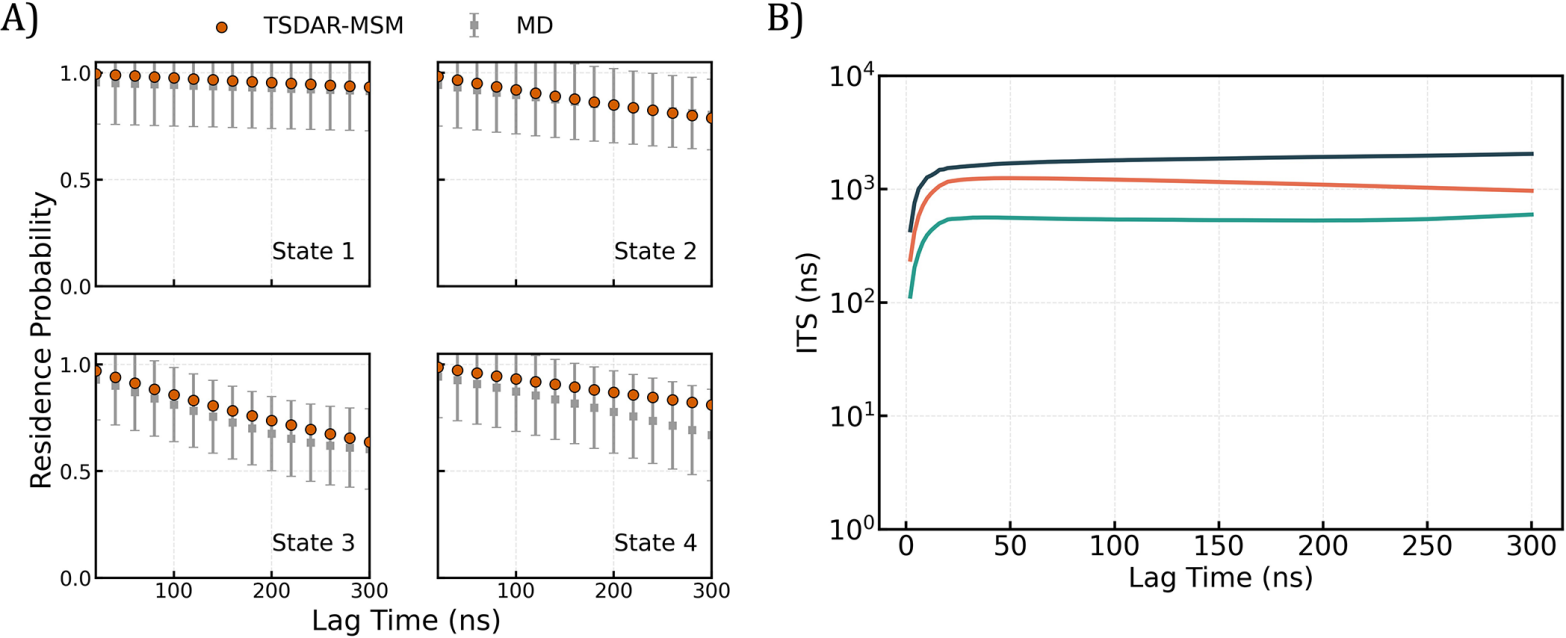
TS-DAR-MSM validation for villin headpiece. (A) Chapman-Kolmogorov test comparing the evolution of state probabilities predicted by the TS-DAR-MSM (orange points) with those observed from MD trajectories (gray points). The error bars are calculated by bootstrapping 160 MD trajectories 50 times with replacement. The agreement between the two shows that the TS-DAR-MSM accurately captures the long-time scale kinetics of the system. (B) Implied timescales plot showing the three dominant relaxation timescales as a function of lag time.

**Figure 13:**
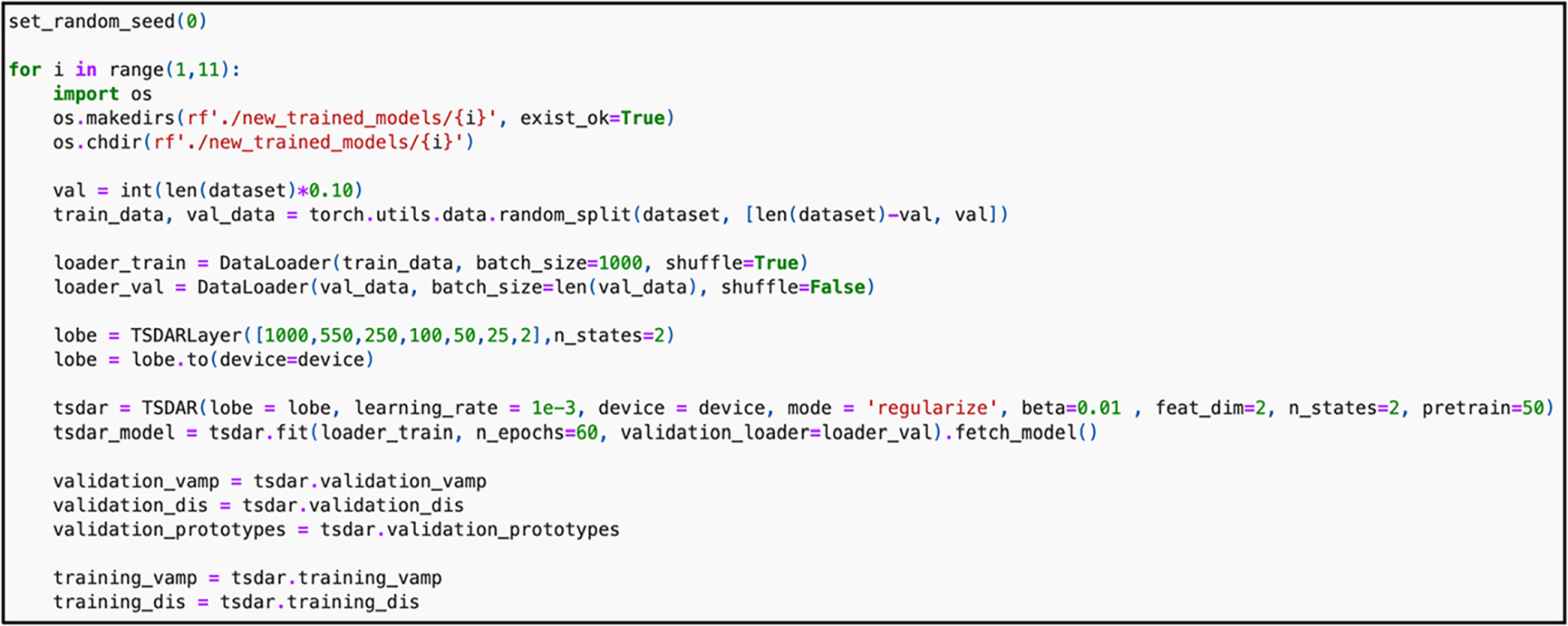
Code to train ten TS-DAR models for protein phosphatase 2A.

To improve the TS-DAR framework, traditional feature selection mechanisms, like spectral oASIS, can be replaced by more advanced approaches like equivariant neural networks.^45^ These networks, which account for the symmetries inherent in molecular systems (e.g., rotational and translational invariance), could offer a more efficient and robust way to capture the most relevant structural features of proteins. Integrating equivariant neural networks into the TS-DAR framework could enhance the overall performance, especially when dealing with large, complex systems. This is because, in these cases, the conformational changes involve high-dimensional or nonlinear features that traditional methods may not capture effectively. The equivariant neural network can directly process the Cartesian coordinates of the protein’s C-α atoms and, therefore, eliminate the need for manual feature selection. Another next step could be to use the collective variables (CVs) derived from TS-DAR latent space representations for enhanced sampling.^46,47^ For example, one could perform metadynamics^48,49^ along the CVs identified by TS-DAR to efficiently sample rare transition events and metastable states. Alternatively, given a pair of initial and final states, the CVs can be transformed into a committor function, connecting these states through a suitable linear combination and enabling a probabilistic estimate of transition likelihood.^50^ Finally, the state assignments obtained through TS-DAR could be used to construct GME-based models, offering a more accurate representation of non-Markovian dynamics in biomolecular systems.^17,20^ Overall, TS-DAR is a powerful tool for studying molecular transitions, and with continued refinement, it can significantly advance our understanding of biomolecular function. This can have far-reaching implications for the design of novel therapeutics.

**Figure 14:**
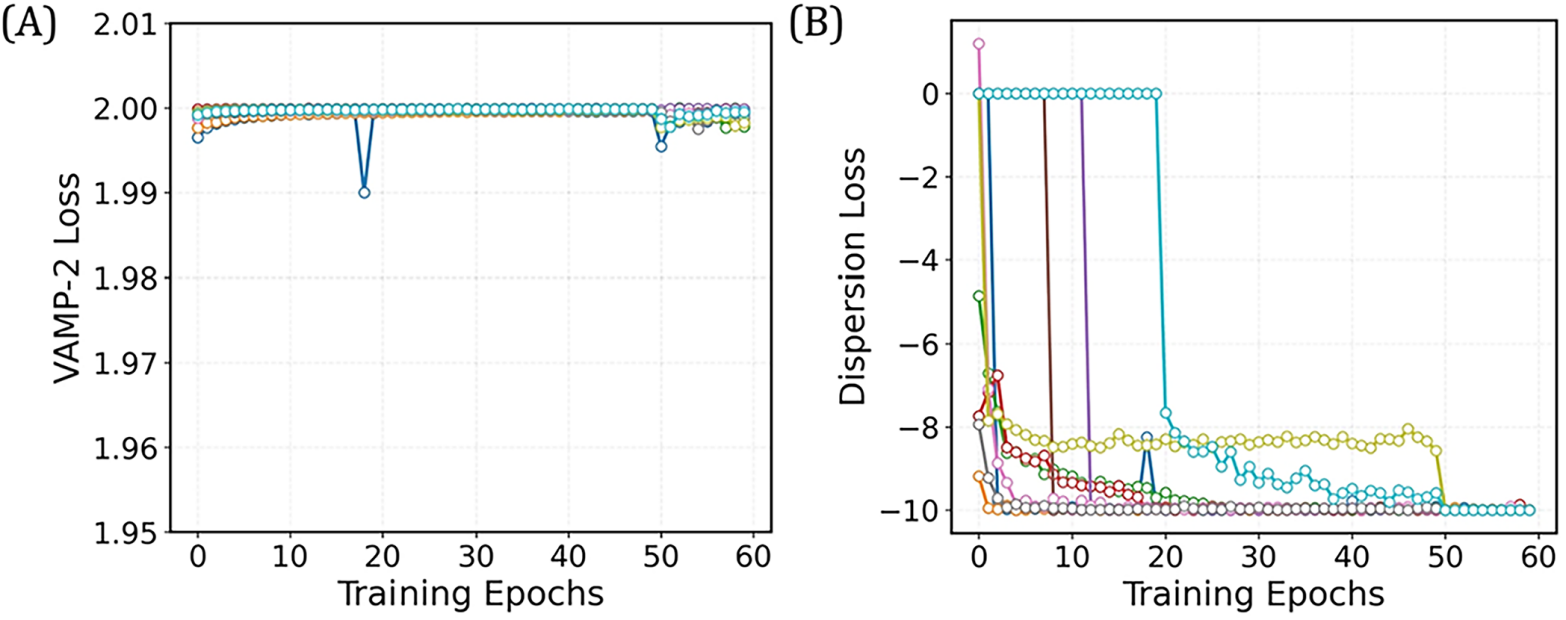
Validation curves for the 2-state TS-DAR model for PP2A model. (A) Validation curve for the VAMP-2 loss. (B) Validation curve for the dispersion loss.

**Figure 15:**
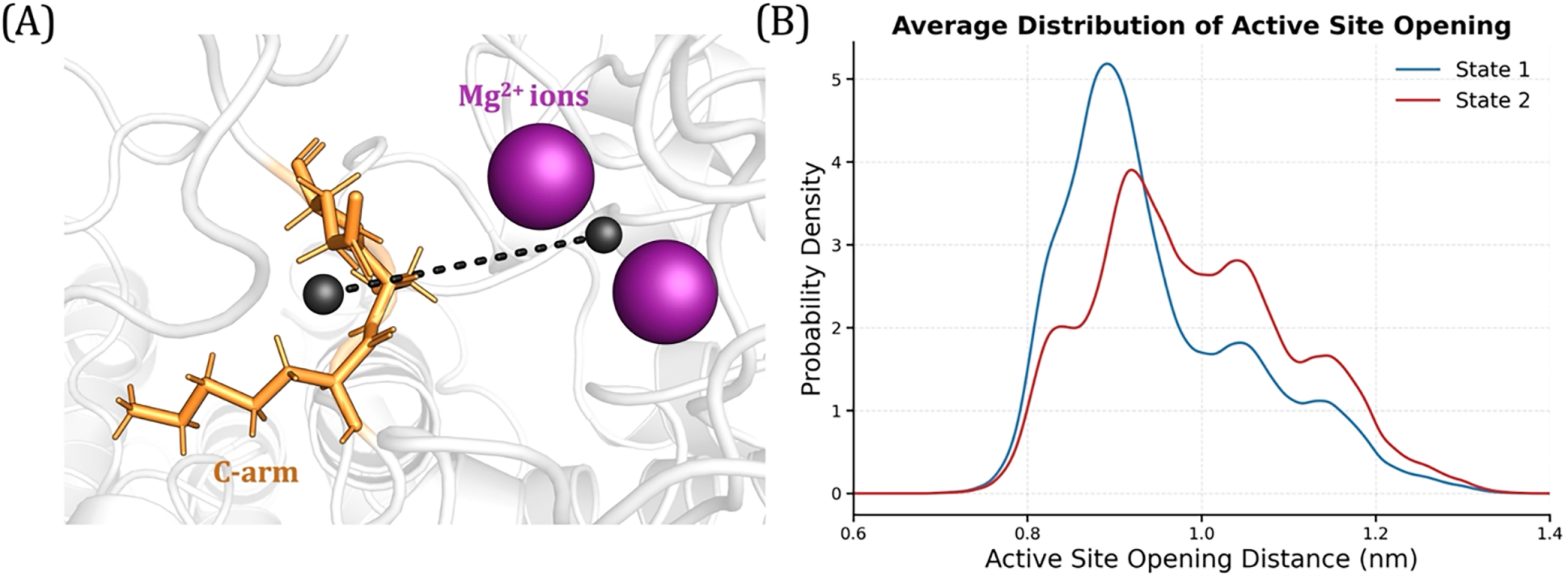
PP2A active site opening. (A) Schematic illustrating the active site opening distance. The distance (black dashed line) was measured between the center of mass (shown as black spheres) of residues 572–574 on the C-arm (colored orange) and the Mg^2+^ ions (colored purple) in the catalytic site. (B) Average probability density distribution of the active site opening distance for state 1 (shown in blue) and state 2 (shown in red).
